# Gut Bacteria-Derived Tryptamine Ameliorates Diet-Induced Obesity and Insulin Resistance in Mice

**DOI:** 10.3390/ijms26031327

**Published:** 2025-02-04

**Authors:** Jongjun Lee, Hye-Rim Jang, Dongjin Lee, Yeonmi Lee, Hui-Young Lee

**Affiliations:** 1 Laboratory of Mitochondria and Metabolic Diseases, Lee Gil Ya Cancer and Diabetes Institute, Gachon University, Incheon 21999, Republic of Korea; ljjsuika@gmail.com (J.L.); herim1126@gachon.ac.kr (H.-R.J.); 2 Department of Health Sciences and Technology, Gachon Advanced Institute for Health Sciences & Technology, Gachon University, Incheon 21999, Republic of Korea; 3 Department of Molecular Medicine, Gachon University College of Medicine, Incheon 21936, Republic of Korea

**Keywords:** tryptamine, gut bacterial metabolite, high-fat diet, obesity, adipose tissue

## Abstract

Tryptophan is an essential amino acid that is metabolized in the intestine by gut bacteria into indole derivatives, including tryptamine. However, little is known about which bacterial tryptophan metabolites directly influence obesity. In this study, we identified tryptamine as a bacterial metabolite that significantly reduced fat mass following the intraperitoneal injection of five bacterial tryptophan end-products in a diet-induced obese mouse model. Interestingly, tryptamine, a serotonin analog, inhibited both lipogenesis and lipolysis in adipose tissue, which was further confirmed in a 3T3-L1 adipocyte cell culture study. Moreover, oral tryptamine supplementation markedly reduced fat mass and improved insulin sensitivity in a long-term, high-fat-diet, pair-feeding model. These studies demonstrate the therapeutic potential of tryptamine, a bacterial tryptophan metabolite, in ameliorating obesity and insulin resistance by directly regulating lipogenesis and lipolysis in white adipose tissue.

## 1. Introduction

The gut microbiota plays a pivotal role in maintaining physiological and metabolic homeostasis in the host [1]. Disruption to the gut microbial ecosystem, termed dysbiosis, has been implicated in the development of obesity [2,3], type 2 diabetes (T2D) [4,5], and chronic inflammatory diseases [6]. Clinical studies have shown that the manipulation of the gut microbiota through probiotic supplementation or fecal microbial transplantation can modulate the host’s energy balance, lipid metabolism, and glycemic control, providing therapeutic benefits for diet-induced obesity, insulin resistance, and T2D [4,7,8,9]. However, the precise mechanisms by which the gut microbiota interacts with host metabolism remain poorly understood.

There is increasing evidence that bacterial metabolites, as functional outputs of the microbiota, are important modulators of host physiology. These small molecules enter the circulatory system and reach concentrations comparable to those of a typical drug dose, influencing host biology by interacting with specific receptors [10,11]. Among these, short-chain fatty acids (SCFAs) and amino acid derivatives have been recognized as key modulators of host metabolism that influence host physiology and disease progression [12,13]. In particular, tryptophan possesses a unique metabolic pathway that is not degraded by the host but relies on the gut microbiota [14,15]. Therefore, gut microbiota have now been recognized not only to influence the primary functions of tryptophan on the gut–brain axis [14,16] and metabolic diseases [14,15,17] but also to produce microbe-specific tryptophan metabolites that are currently gaining attention for their distinct biological roles [15].

Tryptophan, an essential amino acid obtained solely through the diet, plays a crucial role in protein biosynthesis and serves as a precursor for the synthesis of multiple important bioactive compounds [17]. Tryptophan is metabolized via three major pathways: the kynurenine (Kyn), 5-hydroxytryptamine (5-HT; serotonin), and bacterial indole pathways [17,18]. In the gastrointestinal tract, the gut microbiota metabolizes tryptophan into several derivatives, including indole, indole-3-propionic acid (IPA), indole-3-acetic acid (I3A), indole-3-carboxaldehyde (ICA), and tryptamine [18,19]. These metabolites regulate host biological processes such as the immune response, epithelial barrier integrity, and metabolism [18,19,20].

Notably, microbial tryptophan metabolites have shown potential in the treatment of metabolic diseases. For example, indole and I3A ameliorate lipid-induced hepatic steatosis and inflammation in vitro [21] and in vivo [22,23], suggesting their therapeutic potential in non-alcoholic fatty liver disease (NAFLD). However, although these metabolites improve hepatic steatosis and inflammation, they do not reduce body weight or adiposity in a high-fat diet (HFD)-induced obese model [22,23]. This highlights the need to investigate other tryptophan metabolites to determine their anti-obesity potential. To our knowledge, studies on the anti-obesity effects of other microbial tryptophan metabolites in HFD-induced obesity models are lacking.

In this study, we aimed to identify microbial tryptophan metabolites that can combat obesity. Using an HFD-induced obesity model, we examined five bacterial tryptophan metabolites with both intraperitoneal (IP) and oral supplementary routes to evaluate their effects on fat mass and metabolic parameters. We further explored their therapeutic potencies and underlying mechanisms by performing a glucose/insulin tolerance test and indirect calorimetry and cell culture studies. Our findings offer new insights into the potential of microbial tryptophan metabolites as therapeutic agents for obesity and related metabolic disorders.

## 2. Results

### 2.1. Tryptamine Reduces Body Weight and Fat Mass During Short-Term Intraperitoneal Injection Treatment Under HFD Conditions

**Figure 1:**
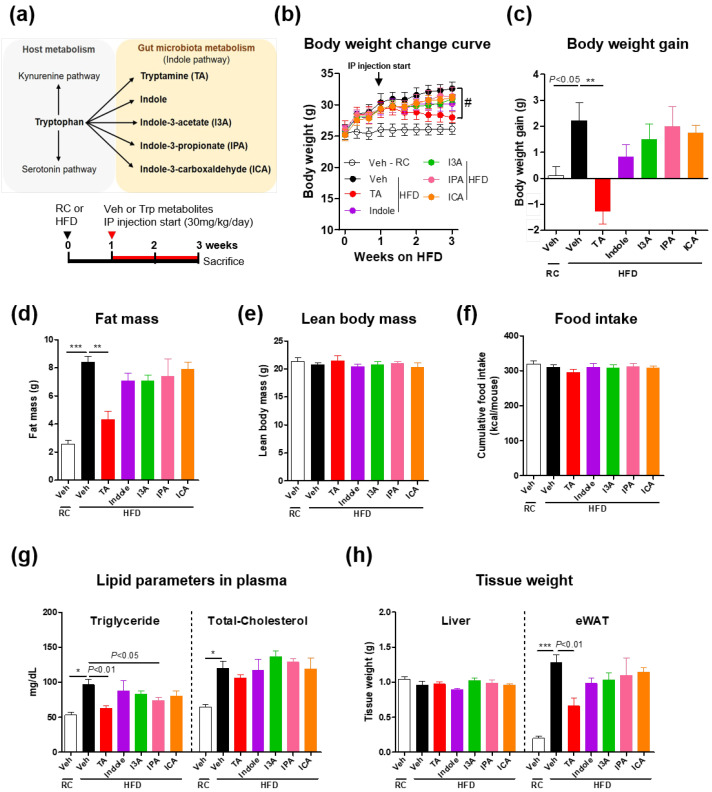
Tryptamine, among other microbial tryptophan metabolites, reduced body weight and fat mass during short-term intraperitoneal injections under high-fat diet conditions. (**a**) Schematic representation of the tryptophan metabolic pathway in the host and gut microbiota (upper) and in vivo study design (bottom). (**b**–**h**) Mice were fed RC or an HFD for three weeks, with vehicle, tryptamine, indole, indole-3-acetate, indole-3-proprionic acid, or indole-3-carboxaldehyde administered daily via IP injection during the final 2 weeks. All groups fasted overnight prior to being sacrificed. (**b**) Body weight change curve for 3 weeks of RC or HFD feeding. (**c**) Final body weight gain, (**d**) fat mass, and (**e**) lean body mass. (**f**) Cumulative food intake for 3 weeks of RC or HFD. (**g**) Lipid parameters in plasma. (**h**) Tissue weight; *n* = 4–5 for each group. Data are presented as the mean ± SEM. # *p* < 0.05 according to the two-way ANOVA. * *p* < 0.05, ** *p* < 0.01, and *** *p* < 0.001 according to the one-way ANOVA. Statistical number was obtained by Student’s *t*-test. RC, regular chow; HFD, high-fat diet; Veh, vehicle; Trp, tryptophan; TA, tryptamine; I3A, indole-3-acetate; IPA, indole-3-propionic acid; ICA, indole-3-carboxaldehyde; IP, intraperitoneal; eWAT, epididymal white adipose tissues; SEM, standard error of the mean.

Tryptophan is ultimately metabolized by the gut bacteria into five indole derivatives: indole, I3A, IPA, ICA, and tryptamine (Figure 1a upper). To identify tryptophan-derived metabolites that directly influence adiposity, we administered short-term IP injections to HFD-fed mice (Figure 1a bottom). Among the five metabolites tested, tryptamine treatment resulted in a dramatic reduction in body weight immediately after the injection (Figure 1b). Notably, weight gain during HFD feeding was negative in the tryptamine group, whereas the other groups exhibited positive weight gain (Figure 1c). The observed weight loss (approximately 3 g) was attributed entirely to a reduction in fat mass (Figure 1d), as measured by ^1^ H-nuclear magnetic resonance, without significant differences in the lean body mass between the groups (Figure 1e). Food intake remained consistent across all groups, ruling out reduced caloric intake as a contributing factor (Figure 1f). Furthermore, plasma triglyceride (TG) levels were significantly lower in the tryptamine-treated group, whereas total cholesterol levels did not differ between the groups (Figure 1g). There was a significant reduction in epididymal white adipose tissue (eWAT) weight in the tryptamine group but no significant difference in liver tissue weight (Figure 1h). These results suggest that tryptamine, a gut bacterial metabolite, has a strong anti-obesity effect by targeting the adipose tissue, independent of the intestinal route.

### 2.2. Tryptamine Treatment Shifts Energy Substrate Preference to Fat and Reduces Locomotor Activity Without Altering Energy Expenditure

**Figure 2:**
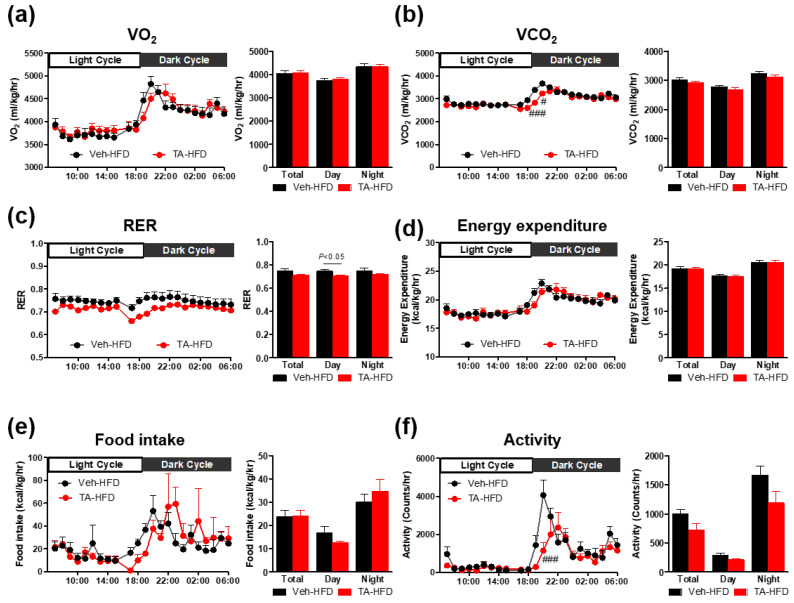
Tryptamine decreased respiratory exchange ratio during short-term intraperitoneal injection administration under high-fat diet conditions. (**a**) Oxygen consumption, (**b**) carbon dioxide production, (**c**) respiratory exchange ratio, (**d**) energy expenditure, (**e**) food intake, and (**f**) activity were measured in mice housed in individual metabolic cages for 48 h; *n* = 7–8 for each group. Data are presented as the mean ± SEM. # *p* < 0.05 and ### *p* < 0.001 according to the two-way ANOVA. Statistical number was obtained by Student’s *t*-test. Veh, vehicle; HFD, high-fat diet; TA, tryptamine; VO_2_, oxygen consumption; VCO_2_, carbon dioxide production; RER, respiratory exchange ratio; SEM, standard error of the mean.

To determine whether the physiological mechanism underlying the anti-obesity effects of tryptamine is associated with changes in whole-body energy expenditure, we performed indirect calorimetry tests on vehicle- and tryptamine-treated HFD-fed mice. To minimize the influence of significant body weight differences, the energy balance was monitored after one week of daily IP injections of tryptamine along with HFD feeding. Oxygen consumption (VO_2_) was not significantly different between the tryptamine-treated and vehicle groups (Figure 2a). Carbon dioxide production (VCO_2_) and respiratory exchange ratio (RER) were significantly lower in the tryptamine-treated mice during the daytime cycle (Figure 2b,c). Whole-body energy expenditure and total food intake remained unchanged between the groups (Figure 2d,e). However, the tryptamine-treated group showed a slight delay in food access, as indicated by the shifting of the food intake curve to the right (Figure 2e). The total locomotor activity was lower in the tryptamine-treated group than in the vehicle-treated group (Figure 2f). These data indicate that tryptamine treatment enhances the preference for fat as a substrate during the inactive day-time phase while maintaining energy expenditure, despite a reduction in locomotor activity and a subtle delay in appetite.

### 2.3. Tryptamine Treatment Directly Inhibits Lipogenesis in Adipose Tissue and 3T3L1 Cells

**Figure 3:**
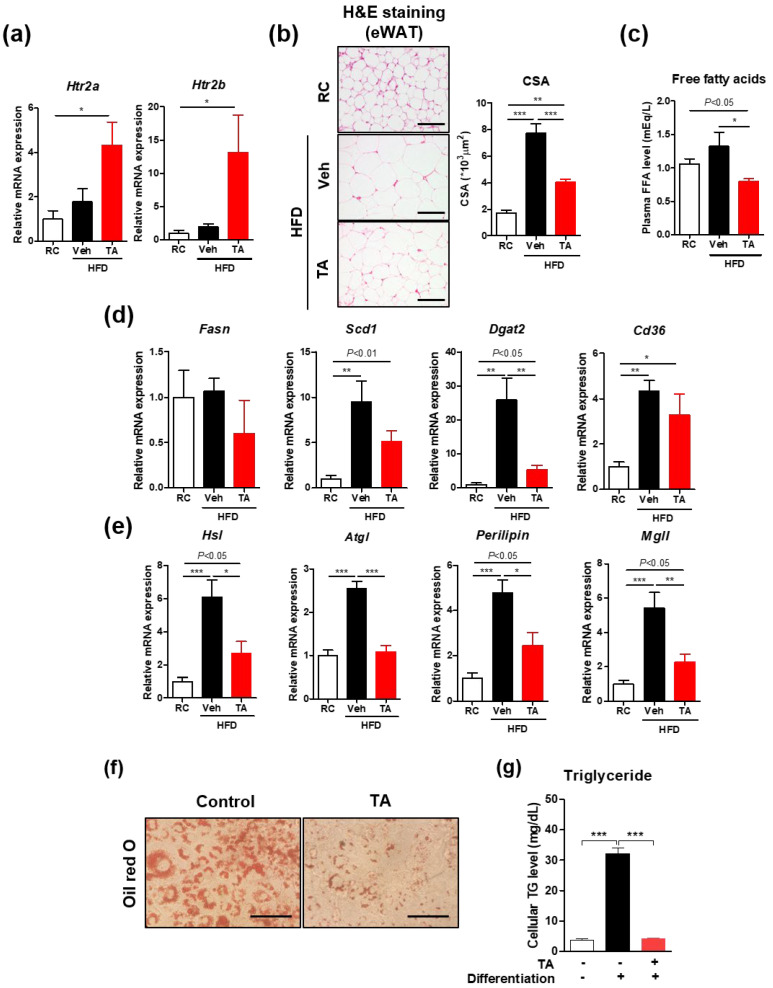
Tryptamine decreased lipogenesis and lipolysis in white adipose tissue during short-term intraperitoneal injections under high-fat diet conditions. (**a**–**e**) Mice were fed RC or an HFD for three weeks, with Veh or TA administered daily via IP injection during the final 2 weeks. Analyses were performed in all groups that fasted overnight prior to being sacrificed. (**a**) *Htr2a* and *Htr2b* mRNA expression in eWAT. (**b**) Representative H&E staining images and cross-sectional area of eWAT. Scale bar = 100 μm. (**c**) Plasma free fatty acids. mRNA expression of genes involved in (**d**) lipogenesis and (**e**) lipolysis in eWAT; *n* = 4–5 for each group. (**f**,**g**) Adipocyte 3T3-L1 cells were incubated with vehicle or TA for 48 h. (**f**) Oil Red O staining. Scale bar = 100 μm. (**g**) Cellular triglyceride level. Data are presented as the mean ± SEM. * *p* < 0.05, ** *p* < 0.01, and *** *p* < 0.001 according to the one-way ANOVA. Statistical number was obtained by Student’s *t*-test. RC, regular chow; Veh, vehicle; HFD, high-fat diet; TA, tryptamine; IP, intraperitoneal; eWAT, epididymal white adipose tissues; *Htr*, 5-hydroxytryptamine; H&E, hematoxylin and eosin; CSA, cross-sectional area; *Fasn*, fatty acid synthase; *Scd1*, stearoyl-CoA desaturase 1; *Dgat2*, diglyceride acyltansferase 2; *Cd36*, cluster of differentiation 36; *Hsl*, hormone-sensitive lipase; *Atgl*, adipose triglyceride lipase; *Mgll*, monoglycerol lipase; TG, triglyceride; SEM, standard error of the mean.

Compared to other microbial tryptophan metabolites, tryptamine has the closest structural similarity to serotonin and interacts with serotonin receptors, specifically the hydroxytryptamine receptor (HTR) [24]. *Htr2a* and *Htr2b* regulate lipid metabolism in the adipose tissue [25], which is typically associated with lipogenesis under fed conditions (*Htr2a*) [26] and lipolysis under fasting conditions (*Htr2b*) [25]. Tryptamine treatment markedly increased the expression of both the receptors (Figure 3a). Interestingly, despite the upregulation of *Htr2a* and *Htr2b*, the tryptamine-treated mice exhibited reduced adipocyte size (Figure 3b) and lower plasma free fatty acid levels than the HFD-fed controls (Figure 3c). Moreover, the expression of lipogenesis- and lipolysis-related genes was mostly higher in the HFD-fed mice than the regular chow (RC)-fed mice (Figure 3d,e). This expression was significantly reduced in the tryptamine-treated mice compared to the HFD-fed mice, suggesting that tryptamine exerts an overall inhibitory effect on lipid metabolism. To confirm the direct effect of tryptamine on lipogenesis, we performed in vitro experiments using 3T3-L1 adipocytes and exposed them for 48 h to tryptamine. Oil Red O staining revealed a marked reduction in lipid droplet formation (Figure 3f), and the subsequent quantification of cellular triglyceride content further demonstrated a significant decrease in lipid accumulation following tryptamine treatment (Figure 3g). Collectively, these findings from both in vivo and in vitro experiments indicate that tryptamine directly inhibits lipid accumulation in adipocytes by modulating serotonin receptor signaling.

### 2.4. Oral Tryptamine Supplementation Sustains Anti-Obesity Effect Under Long-Term HFD Conditions

**Figure 4:**
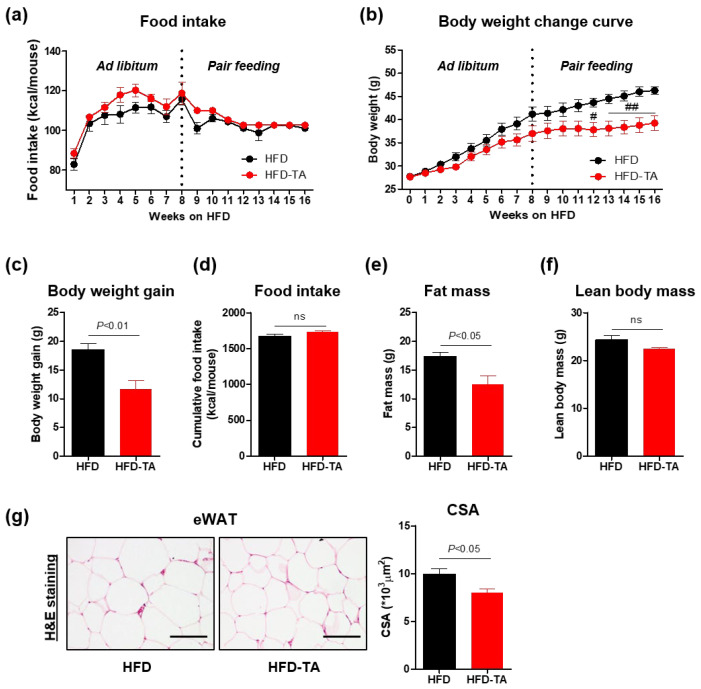
Tryptamine supplementation attenuated HFD-induced obesity under long-term pair-feeding conditions. (**a**–**g**) Mice were fed an HFD or customized HFD containing 0.1% tryptamine for 16 weeks, with ad libitum feeding for the first 8 weeks and pair-feeding for the final 8 weeks. (**a**) Food intake. (**b**) Body weight change curve. (**c**) Body weight gain and (**d**) accumulative food intake over 16 weeks of HFD feeding. (**e**) Final fat mass and (**f**) lean body mass. (**g**) Representative H&E staining images and cross-sectional area of eWAT. Scale bar = 100 μm; *n* = 5–6 for each group. Data are presented as the mean ± SEM. # *p* < 0.05 and ## *p* < 0.01 according to the two-way ANOVA. Statistical number was achieved by a Student’s *t*-test. ns, non-significant; HFD, high-fat diet; TA, tryptamine; H&E, hematoxylin and eosin; eWAT, epididymal white adipose tissues; CSA, cross-sectional area; SEM, standard error of the mean.

Because gut microbial metabolites exert their physiological effects primarily in the intestine, we aimed to determine whether the anti-obesity effects of tryptamine observed with IP injections could also be achieved through oral administration. To address this, we initially designed a long-term experiment of 8 weeks with a customized HFD containing 0.1% tryptamine (1 g/kg HFD). The tryptamine-supplemented mice exhibited a trend toward increased food intake compared to the HFD-fed mice (*p* < 0.1 by Student’s *t*-test at 5 weeks, Figure 4a); however, they showed a tendency toward decreased body weight compared to the HFD-fed mice after 8 weeks on the HFD (*p* < 0.1 by Student’s *t*-test, Figure 4b). Therefore, pair-feeding was introduced from the eighth week onward to rule out the influence of food intake. After 4 weeks of pair-feeding, a significant reduction in weight gain was observed in the tryptamine group compared to the controls (Figure 4b,c) without differences in cumulative food intake during the entire 16-week experimental period (Figure 4a,d). The tryptamine-fed mice showed significantly reduced fat mass (Figure 4e) with no difference in lean body mass (Figure 4f). Histological analysis showed that long-term oral supplementation with tryptamine reduced eWAT cell size (Figure 4g). These results demonstrate that orally administered tryptamine exerts sustained anti-obesity effects under HFD conditions, similarly to the effects observed with IP injection.

### 2.5. Oral Tryptamine Supplementation Improved Glucose Tolerance and Whole-Body Insulin Sensitivity in HFD-Fed Mice

**Figure 5:**
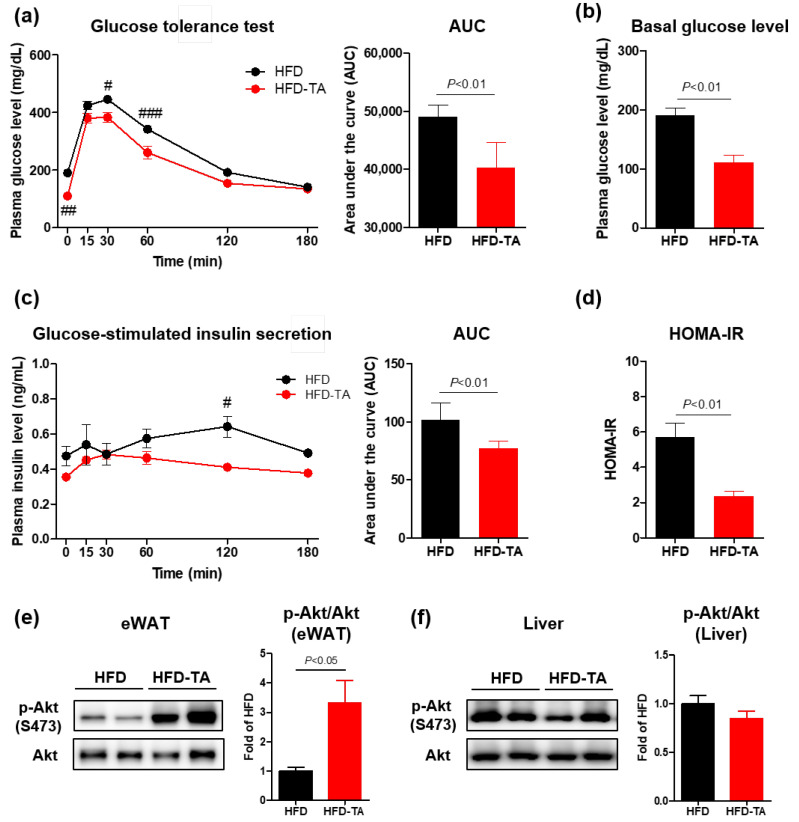
Tryptamine supplementation improved whole-body insulin sensitivity under long-term pair-feeding HFD conditions. (**a**–**d**) Mice were fed an HFD or customized HFD containing 0.1% tryptamine for 16 weeks, with ad libitum feeding for the first 8 weeks and pair-feeding for the final 8 weeks. Analyses were performed in groups that fasted overnight prior to experiments. (**a**) Plasma glucose concentrations (left) and AUC (right) during glucose tolerance test. (**b**) Basal plasma glucose level, (**c**) plasma insulin concentrations (left) and AUC (right), and (**d**) HOMA-IR during the glucose tolerance test. (**e**,**f**) Insulin signaling analyses were performed in the HFD and TA groups after fasting for 6 h. Representative immunoblots of phospho Akt (*p*-Akt on Ser473) and total Akt in (**e**) eWAT and (**f**) liver of HFD and TA groups 30 min after insulin injection; *n* = 5 for each group. Data are presented as the mean ± SEM. # *p* < 0.05, ## *p* < 0.01, and ### *p* < 0.001 according to the two-way ANOVA. Statistical number was obtained by Student’s *t*-test. HFD, high-fat diet; TA, tryptamine; AUC, area under curve; HOMA-IR, homeostatic model assessment of insulin resistance; eWAT, epididymal white adipose tissue; SEM, standard error of the mean.

To determine whether the anti-obesity effects of tryptamine improved glucose metabolism, we performed a glucose tolerance test (GTT) and analyzed insulin signaling in long-term HFD-fed mice. The tryptamine-supplemented mice exhibited lower fasting plasma glucose concentrations and reduced glucose levels following glucose loading during the GTT compared to the controls (Figure 5a,b). Moreover, the fasting plasma insulin levels tended to decrease in the tryptamine-treated mice, and the area under the curve (AUC) of glucose-stimulated insulin secretion was significantly reduced (Figure 5c). Consistently, the homeostatic model assessment of insulin resistance (HOMA-IR) was significantly reduced in the tryptamine-supplemented group compared to the control groups (Figure 5d), indicating improved whole-body insulin sensitivity.

To further explore tissue-specific improvements in insulin signaling, we measured Akt phosphorylation at Ser473 in the adipose tissue and liver 30 min after an insulin injection. Akt phosphorylation was significantly increased in the eWAT (Figure 5e), but not in the liver (Figure 5f), of the tryptamine-supplemented mice, suggesting that tryptamine exerts its effects specifically on adipose tissue, consistent with the findings of the IP injection study. These results demonstrate that tryptamine improves whole-body insulin sensitivity in HFD-fed mice by enhancing insulin signaling in the adipose tissue.

## 3. Discussion

While previous studies have focused on microbial tryptophan metabolites, such as indole and I3A, for their roles in alleviating hepatic steatosis, inflammation, and glucose metabolism, their effects on diet-induced obesity have been minimal. For example, the oral administration of indole and IP injection of I3A have been shown to improve hepatic steatosis and inflammation in mice under long-term HFD conditions [22,23]. However, neither treatment significantly reduced HFD-induced weight gain [22,23]. Similarly, ICA ameliorates hepatic inflammation and fibrosis in a 3,5-diethoxycarbonyl-1,4-dihydrocollidine-induced primary sclerosing cholangitis model [27], and indole stimulates enteroendocrine L cells to produce glucagon-like peptide-1 (GLP-1), enhancing insulin secretion by pancreatic β cells in vitro [28]. Additionally, IPA lowers circulating glucose and insulin levels in a IPA-rich regular chow diet [29], and long-term HFD feeding reduces tryptamine levels in the liver, cecum, and serum of mice [21]; however, none of these have successfully demonstrated anti-obesity effects.

The liver plays a key physiological role as a major fat distributor by delivering dietary lipids to peripheral tissues in the form of very low-density lipoprotein and converting glucose to lipids via de novo lipogenesis [30]. Considering these roles of the liver, it is puzzling that substances that improve fatty liver function do not exhibit anti-obesity effects. This suggests that opposing mechanisms of adipose tissue or appetite regulation may counteract these effects, highlighting the need for further research on their anti-obesity potential. Therefore, in this study, we demonstrated, for the first time, that tryptamine, a microbial tryptophan metabolite, exerts significant anti-obesity effects under both short- and long-term HFD feeding conditions. Among the five microbial tryptophan end-product metabolites, only tryptamine significantly reduced fat mass following short-term IP administration in the HFD-fed mice, and it inhibited lipogenesis in the adipose tissue and adipocytes. This effect persisted under long-term HFD feeding, where tryptamine improved insulin signaling in adipose tissues, as reflected in whole-body insulin sensitivity. These findings underscore the unique potential of tryptamine as a microbial metabolite for combating diet-induced obesity and insulin resistance by differentiating it from other tryptophan metabolites that primarily target the liver.

Mechanistically, the effects of tryptamine on adipose tissue can occur through two main pathways: the aryl hydrocarbon receptor (AhR) and the serotonin receptor (HTR). Microbial tryptophan metabolites are well-known activators of AhR, particularly in the gut [15,18], where AhR activation enhances the intestinal barrier function and stimulates GLP-1 secretion, leading to improved glucose metabolism and hepatic steatosis [31]. Although gut-specific AhR activation has been associated with metabolic benefits, its activation in adipose tissue has shown opposing effects. Adipose tissue-specific AhR activation exacerbated insulin resistance, whereas adipose tissue-specific AhR deletion improved insulin sensitivity [32]. All five microbial tryptophan end-product metabolites evaluated in this study activated AhR [15]. However, only tryptamine significantly reduced eWAT weight under HFD feeding conditions, suggesting that its anti-obesity effects are mediated through mechanisms other than AhR signaling. However, the structural similarity between tryptamine and serotonin suggests its potential to interact with HTR [24]. Serotonin receptors, particularly HTR2A and *2*B, regulate lipid metabolism in the adipose tissue. The expression of serotonin receptors has been reported to be increased in the WAT of individuals with obesity [25]. *Htr2a* induces lipogenesis under fed conditions [26], whereas *Htr2b* increases lipolysis under fasting conditions [25], suggesting that inhibiting both receptors could serve as a strategy to improve insulin resistance in adipose tissue. Interestingly, in this study, tryptamine not only reduced eWAT mass, but also upregulated the expression of *Htr2a* and *2b*. Despite its upregulation, tryptamine inhibited the expression of *Htr2* downstream genes related to both lipogenesis and lipolysis. Although the exact mechanism remains to be elucidated, the metabolic effect of tryptamine on adipose tissue likely occurs through *Htr2* pathways. These findings highlight a novel mechanism by which tryptamine reduces WAT weight under HFD conditions, potentially through the inhibition of lipid metabolism via serotonin receptors. Further studies are required to elucidate the precise mechanisms underlying this inhibition.

Existing studies on tryptamine, a gut-derived serotonin-like metabolite, have yielded conflicting results. Elevated tryptamine levels have been linked to glucose intolerance in patients with T2D, and acute IP administration impaired insulin sensitivity in mice [33]. Conversely, long-term HFD feeding reduces tryptamine levels across tissues, highlighting its potential role in metabolic health [21]. However, these studies are limited by their short-term analyses, non-physiological administration routes, and lack of focus on adipose tissue regulation. In this study, we addressed these gaps by demonstrating that the long-term oral administration of tryptamine, which mimics its natural gut origin, significantly reduces HFD-induced body weight gain and fat mass. Importantly, unlike previous microbial metabolites that showed little to no effect on adiposity, tryptamine directly modulated lipid metabolism in WAT. The pair-feeding experiments revealed that the weight-reducing effects of tryptamine were independent of food intake. In the present study, the tryptamine-induced increase in food intake occurred only when it was administered orally. The oral route of tryptamine administration suggests that it may exert its unique roles in the gut, such as accelerating gastrointestinal transit by stimulating anion-dependent fluid secretion in the proximal colon via the activation of epithelial HTR4 receptors [34]. This novel mechanistic insight highlights the dual role of tryptamine in appetite regulation and lipid metabolism, making it a promising candidate to combat HFD-induced obesity.

However, this study has several limitations. (1) Although tryptamine exhibits significant anti-obesity effects, the precise molecular mechanisms underlying its regulation of lipid metabolism in WAT remain unclear, particularly its interaction with the HTR2A and HTR2B receptors. (2) As a therapeutic agent, tryptamine inhibits lipogenesis and is beneficial for both obesity and insulin resistance. The suppression of adipose lipolysis can reduce the amount of fatty acids and glycerol entering the liver, thereby protecting against hepatic insulin resistance. However, it can also cause adipose tissue expansion and exacerbate obesity. Future drug development must consider the dual effects of tryptamine and ensure precise regulation. (3) Additionally, the increased food intake observed with oral tryptamine administration, although mitigated under pair-feeding conditions, raises questions regarding its long-term effects on energy homeostasis and appetite regulation. Future studies should explore the dose-dependent effects of tryptamine and evaluate its safety profile in chronic conditions. Furthermore, the potential crosstalk between gut-derived tryptamine and other metabolic tissues, such as the liver and brain, warrants further investigation to fully elucidate its systemic metabolic effects.

In conclusion, our findings demonstrate the therapeutic potential of tryptamine, a bacterial tryptophan metabolite, in ameliorating obesity and insulin resistance by regulating lipogenesis and lipolysis in adipose tissue. This study identified tryptamine as a promising anti-obesity agent that uniquely combines adipose tissue-specific and whole-body metabolic effects.

## 4. Materials and Methods

### 4.1. Animal Study

Male C57BL/6J mice were purchased from Central Laboratory Animal Inc. (Seoul, Korea) and were housed in a specific pathogen-free facility under controlled temperature (22 ± 1 °C), humidity (55 ± 10%), and lighting (12 h light/dark cycle). The mice had ad libitum access to water and one of the following diets—60% HFD (D12492, Research Diets, New Brunswick, NJ, USA) or RC diet (5053, Labdiet, St. Louis, MO, USA)—except in the pair-feeding study. Tryptophan metabolites, including indole, TA, I3A, IPA, and ICA, were obtained from Sigma-Aldrich (St Louis, MO, USA).

Two animal studies were performed based on the HFD duration and the route of metabolite administration. A short-term HFD study was conducted to identify microbial tryptophan metabolites that influence adiposity. After a one-week acclimation period, the mice were fed either RC or HFD for 3 weeks. The mice on the HFD were grouped with their average body weights matched (*n* = 4~5 per group), and tryptophan microbial metabolites (30 mg/kg/day) were IP injected daily during the final 2 weeks. A long-term HFD study was conducted to evaluate the chronic oral effects of tryptamine on diet-induced obesity and insulin resistance. The TA-containing HFD was customized at a concentration of 1 g tryptamine per 1 kg HFD and was obtained from Saeronbio Inc. (Uiwang, Korea). Mice were grouped with their average body weights matched and fed an HFD (*n* = 5) or tryptamine-containing HFD (*n* = 6) for 16 weeks. For the first 8 weeks, they were provided food ad libitum, and for the final 8 weeks, pair-feeding was conducted to ensure equal food intake between the two groups; equal caloric intake was ensured, and pair-feeding was performed through the daily measurement of food intake.

Body weight and food intake were monitored on a weekly basis for all mice. After overnight fasting, the mice were euthanized following anesthesia with 2% isoflurane gas, and tissues were harvested for further analysis. All animal experimental procedures were approved by the Institutional Animal Care and Use Committee of Gachon University.

### 4.2. Body Composition and Basal Energy Balance

Body composition, including lean body mass and fat mass, was measured by ^1^H-nuclear magnetic resonance spectroscopy (BioSpin; Bruker, Billerica, MA, USA) before fasting. The basal energy balance, including oxygen consumption (VO_2_), carbon dioxide production rate (VCO_2_), respiratory exchange ratio (RER), energy expenditure, food intake, and activity, were measured using a Comprehensive Laboratory Animal Monitoring System (CLAMS; Columbus Instruments, Columbus, OH, USA) after one week of daily IP injections with either a vehicle (*n* = 8) or tryptamine (*n* = 7). Measurements were conducted over 72 h, including 24 h of acclimation followed by 48 h of data acquisition, as previously described [35].

### 4.3. Plasma Lipid Parameters

After anesthesia, blood samples were collected by cardiac puncture from overnight-fasted mice in the short-term HFD study (*n* = 4–5 per group), and plasma was obtained by centrifugation for 20 min at 3000× *g*. Plasma triglyceride, total cholesterol, and free fatty acid levels were measured using a Cobas c111 analyzer (Roche Diagnostics, Rotkreuz, Switzerland).

### 4.4. Hematoxylin and Eosin Staining and Adipocyte Cross-Sectional Area Analysis

The preparation and staining of histopathological eWAT samples obtained from overnight-fasted mice in the short-term (*n* = 4–5 per group) and long-term HFD studies (*n* = 5–6 per group) were performed at the core facility of the Institutional Animal Care and Use Committee at Gachon University. Briefly, formalin-fixed eWAT samples were washed with tap water, dehydrated in an ethyl alcohol–xylene series, and embedded in paraffin. Paraffin sections were stained with hematoxylin and eosin (H&E). Five images were randomly captured at 200× magnification using an OLYMPUS CX31 biological microscope and Zen 2.3 software (Carl Zeiss Microscopy, Oberkochen, Germany), and the adipocyte cross-sectional area (CSA) was determined using software ImageJ 1.47t (National Institutes of Health, Bethesda, MD, USA).

### 4.5. Intraperitoneal Glucose Tolerance Test (ipGTT) and Insulin Signaling Analysis

The mice were maintained on an HFD and TA-containing HFD for 16 weeks, with the groups consisting of 5 mice each. Mice were fasted for 16 h and IP injected with 20% glucose at a 1.5 g/kg concentration, and blood was obtained from the tail vein 0, 15, 30, 60, 120, and 180 min after glucose administration. Plasma glucose levels were measured using a GM9 Glucose Analyzer (Analox Instruments, Stourbridge, UK), and the glucose-stimulated insulin levels were measured using a Mouse Ultrasensitive Insulin ELISA, according to the manufacturer’s protocol (80-INSMSU-E01, ALPCO, Salem, NH, USA). To analyze insulin resistance, the homeostatic model assessment for insulin resistance (HOMA-IR) was calculated using the following formula: fasting glucose (mg/dL) × fasting insulin (mU/L)/405 [36]. For insulin signaling analysis, the mice were maintained on an HFD and TA-containing HFD for 16 weeks (*n* = 5 per group) and IP injected with 0.75 U/kg insulin (Eli Lilly and Company, Indianapolis, IN, USA) after 6 h fasting. The eWAT and liver tissues were collected 30 min after the insulin injection and stored at –80 °C until Western blotting for the analysis of protein expression.

### 4.6. Immunoblotting

Snap frozen tissues from eWAT and the liver were used for protein extraction. Once the tissues were homogenized in liquid nitrogen, RIPA lysis buffer (Cell Signaling Technology, Danvers, MA, USA) supplemented with a protease inhibitor cocktail (Thermo Fisher Scientific, Waltham, MA, USA) and a phosphatase inhibitor cocktail (Sigma-Aldrich, St. Louis, MO, USA) was added. Then, lysates were homogenized and incubated on ice for 30 min. The samples were centrifuged for 20 min at 16,000× *g*, and the supernatants were used for immunoblotting. The total protein concentration was determined using the Bradford assay reagent. Antibodies against Akt (9272S, Cell Signaling Technology, Danvers, MA, USA) and phospho-Akt (4060S, Cell Signaling Technology, Danvers, MA, USA) were used at a 1:1000 dilution. Peroxidase-conjugated goat anti-rabbit IgG (ab6721; Abcam, Cambridge, UK) was used as the secondary antibody at a 1:5000 dilution.

### 4.7. Quantitative Real-Time PCR

Total RNA was extracted from snap frozen tissues of animals that fasted overnight using the TRIzol Reagent (Thermo Fishers Scientific, Waltham, MA, USA). RNA was quantified at 260 nm/280 nm using a NanoDrop 2000C spectrophotometer (Thermo Fisher Scientific, Waltham, MA, USA). The RNA was reverse transcribed using a TOPscript TM RT DryMIX kit according to the manufacturer’s protocol (Enzynomics, Daejeon, Korea). Real-time PCR was performed using the Applied Biosystems 7300 Real-Time PCR system (Thermo Fishers Scientific, Waltham, MA, USA). The primer sequences used in this study are listed in Supplementary Table S1.

### 4.8. Adipogenesis and Oil Red O Staining in 3T3-L1 Cells

The 3T3-L1 murine preadipocyte cell line (ATCC, Manassas, VA, USA) was cultured at 37 °C under 5% CO_2_ in Dulbecco’s Modified Eagle’s Medium (DMEM) with 100 U/mL of penicillin, 100 μg/mL streptomycin, and 10% (*v*/*v*) heat-inactivated calf serum (Thermo Fisher Scientific, Waltham, MA, USA). For differentiation, 3T3-L1 cells were treated with DMEM containing 10% fetal bovine serum (FBS), 100 U/mL of penicillin, 100 μg/mL of streptomycin, 0.5 mM of 3-isobutyl-1-methylxanthine (IBMX), 1 μM of dexamethasone, and 10 μg/mL of insulin solution for 2 d. After the initial differentiation period, the cells were switched to a maturation medium consisting of DMEM supplemented with 10% FBS, 100 U/mL of penicillin, 100 μg/mL of streptomycin, 10 μg/mL of insulin, and 125 μM of tryptamine for 48 h. Oil Red O staining and cellular TG measurements were performed in duplicate across three independent experiments. For cell imaging purposes, the lipid droplets were stained with Oil Red O (Sigma-Aldrich, St. Louis, MO, USA) for 30 min after formalin fixation according to the manufacturer’s protocol. Images were acquired using an inverted microscope (Nikon ESCLIPSE TS100; Nikon Corporation, Tokyo, Japan). The cellular TG content was measured using a TG Quantification Assay Kit (Abcam, Cambridge, UK) according to the manufacturer’s protocol.

### 4.9. Statistical Analysis

All data were expressed as the mean ± SEM. The significance of the differences in the mean values between the two groups was evaluated using a two-tailed unpaired Student’s *t*-test. More than three groups were evaluated using a one-way or two-way ANOVA followed by Bonferroni’s post hoc analysis, using GraphPad Prism software (version 5.0.1), unless otherwise specified.

## Data Availability

Data is contained within the article and Supplementary Materials.

## Supplementary Materials

The following supporting information can be downloaded at: https://www.mdpi.com/article/10.3390/ijms26031327/s1.
