# Supplementary material for: Gut Bacteria-Derived Tryptamine Ameliorates Diet-Induced Obesity and Insulin Resistance in Mice
Source: Int J Mol Sci. 2025 Feb 4;26(3):1327. doi: 10.3390/ijms26031327 (PMC11818187; doi:10.3390/ijms26031327)
Supplement: Supplementary file 1 [file ijms-26-01327-s001.zip › ijms-3416096-supplementary.pdf]

Supplementary Table S1. The primer list used for qPCR in this study.

Name	Source	Forward (5'→3')	Reverse (5'→3')
Htr2a	mouse	TAGCCGCTTCAACTCCAGAACC	AAGACCTTCGAATCATCCTGTAGCC
Htr2b	mouse	CGGGCTACTGCATTCATCAAGA	AGCTCACAGGTGACATTGTGTGG
Fasn	mouse	GGAGGTGGTGATAGCCGGTAT	TGGGTAATCCATAGAGCCCAG
Scd1	mouse	AGACGTCTGGAGGAACATCATT	GCTTGTAGTACCTCCTCTGGAAC
Dgat2	mouse	GCGCTACTTCCGAGACTACTT	GGGCCTTATGCCAGGAAACT
Cd36	mouse	ATTGGTCAAGCCAGCT	TGTAGGCTCATCCACTAC
Hsl	mouse	GGGAGCACTACAAACGCAAC	CAGAGACGACAGCACCTCAA
Atgl	mouse	CGCCTTGCTGAGAATCACCAT	AGTGAGTGGCTGGTGAAAGGT
Perilipin	mouse	GAAGCATCGAGAAGGTGGTAGA	GCATGGTGTGTCGAGAAAGAG
Mgll	mouse	CGGACTTCCAAGTTTTTGTCAGA	GCAGCCACTAGGATGGAGATG
CycloA	mouse	CAAGACTGAATGGCTGGATG	TGGTGATCTTCTTGCTGGTC
